# Supplementary material for: Optimized Treatment Schedules for Chronic Myeloid Leukemia
Source: PLoS Comput Biol. 2016 Oct 20;12(10):e1005129. doi: 10.1371/journal.pcbi.1005129 (PMC5072565; doi:10.1371/journal.pcbi.1005129)
Supplement: S1 Text — (PDF) [file pcbi.1005129.s001.pdf]

Supporting Information: Optimized Treatment Schedules for Chronic Myeloid Leukemia

Qie He[1*], Junfeng Zhu[1], David Dingli[2], Jasmine Foo[3**], Kevin Zox Leder[1***],

1 Department of Industrial and Systems Engineering, University of Minnesota, Minneapolis, MN, USA
2 Department of Hematology, Mayo Clinic, Rochester, MN, USA
3 Department of Mathematics, University of Minnesota, Minneapolis, MN

*qhe@umn.edu, **jyfoo@math.umn.edu, ***lede0024@umn.edu,

A: Parameter Selection

In this section we describe the model parametrization for the examples shown above. A major source of our parameters is the work [1] which statistically fit a hierarchical differential equation model (similar to equation (1) of the main text) to time series data of CML patents undergoing TKI therapy. All birth rates and death rates below are in the units of cells per day.

Stem cell kinetics

- Density dependence parameters ϕ_i of type i stem cell, for each i. We have $\phi_i = 1/(1 + p_i \sum_{i=1}^{n} x_{1,i}(t))$, with $p_1 = (b^0_{1,1}/d^0_{1,1} - 1)/K_1$, $p_2 = (b^0_{1,2}/d^0_{1,2} - 1)/K_2$, and $p_i = p_2$ for $i \geq 3$. The values of K_1 and K_2 are given in Section "Initial cell populations at diagnosis".
- The birth rates $b^j_{1,i}$. The estimates $b^j_{1,1} = 8.00 \times 10^{-3}$ and $b^j_{1,i} = 1.00 \times 10^{-2}$ for any cell type $i \geq 2$ and drug j. The value 1.00×10^{-2} is used in [1] for the birth rate of leukemic stem cells without drug. We further assume that this value remains the same under any therapy, which is different from [1].
- The death rates $d^j_{1,i}$. The estimate $d^j_{1,i} = 5.00 \times 10^{-4}$ for any i and j, from [1].

Progenitor cell kinetics

- The death rates $d^j_{2,i}$. The estimates $d^1_{2,i} = 2.80 \times 10^{-3}$ and $d^2_{2,i} = 5.30 \times 10^{-3}$ for any i, from [1]. The death rate of leukemic progenitor cells under high-dose imatinib (800 mg/day) is 3.50×10^{-3} in [1]. We consider imatinib with regular dose (400 mg/day) in this paper, so we set $d^3_{2,i} = 3.5 \times 10^{-3} \div 2 = 1.75 \times 10^{-3}$ for any i. Note the death rates are the same across all cell types with the same therapy, but vary with different therapies. In addition, we set the death rate of normal progenitor cells $d^0_{2,i} = \min\{d^1_{2,i}, d^2_{2,i}, d^3_{2,i}\} = 1.75 \times 10^{-3}$ for any i.
- The differentiation rates $b^j_{2,i}$.
  - For normal cells, $b^j_{2,1} = 0.350$ for any j. The estimate is from [1]

- For wild type, $b^0_{2,2} = 2b^j_{2,1} = 0.700$, $b^1_{2,2} = b^0_{2,2}/400 = 1.75 \times 10^{-3}$, $b^2_{2,2} = b^0_{2,2}/200 = 3.50 \times 10^{-3}$, and $b^3_{2,2} = b^0_{2,2}/400 = 1.75 \times 10^{-3}$. All estimates are from [1].
- For mutants, the differentiation rates are listed in Table 1. Since there are little *in vivo* data available in the literature related to leukemic mutant birth rate, our estimation is based on *in vitro* data for these mutants, in particular the IC50 values. We use a piecewise linear interpolation to estimate the differentiation rates, based on the relative IC50 values of mutants under nilotinib, dasatinib, and imatinib reported in [2]. For sensitive or moderately resistant mutants (the relative IC50 value is less than or equal to 4), the differentiation rate of mutant i is estimated using the linear interpolation

$$b^j_{2,i} = \text{relative IC50 value of mutant } i \text{ under drug } j \times b^j_{2,2}.$$

For resistant mutants (the relative IC50 value is between 4.01 and 10.0), the differentiation rate is estimated with the following linear interpolation:

$$b^j_{2,i} = 0.9b^4_{2,2} + \frac{0.1b^4_{2.2}}{10.0 - 4.01}(\text{relative IC50 value of mutant } i \text{ under drug } j - 4.01).$$

Thus if the relative IC50 value for a resistant mutant is 4.01, then its differentiation rate is 90% of the differentiation rate of the WT cell without any drug (0.700 per day); if the relative IC50 value for a resistant mutant is 10.0, then its differentiation rate is equal to the birth rate of the WT progenitor cell without drug. For highly resistant mutants (the relative IC50 is larger than 10.0), we set its differentiation rate to the differentiation rate of the WT progenitor cells without drug.

Table 1. The differentiation rate of mutant progenitor cells under three drugs

	E255K	E255V	F317L	M351T	Y253F	V299L
Nilotinib ($b^1_{2,i}$)	0.661	0.700	3.89×10^{-3}	7.70×10^{-4}	5.65×10^{-3}	2.35×10^{-3}
Dasatinib ($b^2_{2,i}$)	0.649	0.0120	0.635	3.08×10^{-3}	5.53×10^{-3}	0.684
Imatinib ($b^3_{2,i}$)	0.654	0.700	4.55×10^{-3}	3.08×10^{-3}	6.27×10^{-3}	2.70×10^{-3}

Differentiated cell kinetics

- The death rate $d^j_{3,i}$. The estimates $d^1_{3,i} = 0.0442$ and $d^2_{3,i} = 0.0394$ for any i, from [1]. The death rate of leukemic differentiation cells under high-dose imatinib (800 mg/day) is 0.0550 in [1]. We consider imatinib with regular dose (400 mg/day) in this paper, so we set $d^3_{3,i} = 0.0550/2 = 0.0275$ for any i. In addition, $d^0_{3,i} = \min\{d^1_{3,i}, d^2_{3,i}, d^3_{3,i}\} = 0.0275$ for any i.
- The differentiation rates $b^j_{3,i}$.
  - For normal cells, $b^j_{3,1} = 5.50$ for any j. The estimate is from [1].
  - For wild type, $b^0_{3,2} = 1.5b^0_{3,1} = 8.25$, $b^1_{3,2} = b^0_{3,2}/600 = 0.0138$, $b^2_{3,2} = b^0_{3,2}/300 = 0.0275$, and $b^3_{3,2} = b^0_{3,2}/600 = 0.0138$. All estimates are from [1].
  - For the mutant, if it is sensitive or moderately resistant to drug j (the relative IC50 value is less than or equal to 4), then $b^j_{3,3} = b^j_{2,3} \times b^j_{3,2}/b^j_{2,2}$, for $j = 1, 2, 3$; otherwise $b^j_{3,3} = b^j_{2,3} \times b^4_{3,2}/b^4_{2,2}$, for $j = 1, 2, 3$.

Terminally differentiated cell kinetics

Using the estimates from [1], we set the differentiation rates $b^j_{4,i} = 100$ and death rates $d^j_{4,i} = 1.00$ for any i and j.

Initial cell populations at diagnosis

The normal marrow output in an adult is approximately 3.50×10^{11} cells per day [3]. To achieve this equilibrium condition, we set $K_1 = 8.75 \times 10^4$ in differential equations from equation (1) of the main text with parameters described in Sections "Stem cell kinetics" to "Terminally differentiated cell kinetics" and in the absence of leukemic cells. To obtain an estimate of K_2, we assume that diagnosis of CML occurs once the leukemic cell burden reaches a threshold of 10^{12} cells [4], and that the differential equations in equation (1) of the main text have parameters described in Sections "Stem cell kinetics" to "Terminally differentiated cell kinetics" and start with $K_1 = 8.75 \times 10^4$ normal stem cells, one wild-type leukemic stem cell, and no other cells. We set $K_2 = 3 \times 10^6$ so that the patient is diagnosed with CML around 78 months (6.5 years) after the first leukemic stem cell arises. At diagnosis, the normal stem cell, progenitor cell, differentiated cell, and terminally differentiated cell populations are 7.34×10^4, 1.61×10^7, 3.24×10^9, and 3.24×10^{11}, respectively; the leukemic stem cell, progenitor cell, differentiated cell, and terminally differentiated cell populations are 2.95×10^5, 4.07×10^7, 1.08×10^{10}, and 1.08×10^{12} respectively. These are used as the initial cell populations for a patient diagnosed with CML.

ANC kinetics

- We require that the patient's ANC cannot fall below $L_{anc} = 1000/\text{mm}^3$. The normal range of the ANC is 1500 to 8000/mm^3. We assume that the normal level of ANC is $U_{anc} = 3000/\text{mm}^3$, and the patient's initial ANC is 3000/mm^3.

- We initially set the monthly decrease rates of ANC to be $d_{anc,1} = 350/\text{mm}^3$ under nilotinib, $d_{anc,2} = 300/\text{mm}^3$ under dasatinib, and $d_{anc,3} = 250/\text{mm}^3$ under imatinib. For imatinib, in [5] it was observed that the median time until the first neutropenia event was 74 days. Given the large range of possible ANC values (1500-8000) at the start of the trial this gives a range of 200/mm^3 to 2800/mm^3 for feasible ANC decrease rates under imatinib. For nilotinib, the reference [6] observes that nilotinib has a higher risk of adverse hematological events than imatinib. Furthermore [6] observes a median time of 24 days until myelosuppresion events. Given that these events may have included lower-grade hematological events we assumed that the median time was an underestimate for the time until neutropenia and thus chose $d_{anc,1} = 350/\text{mm}^3$. We were unable to find any published data on the median time until neutropenia during dasatinib therapy. However, it has been observed that dasatinib induces grade 3 and 4 neutropenias at a higher rate than imatinib [7]. Based on this we initially set dastinib to have a toxicity in between imatinib and nilotinib, but then also considered the scenario where dasatinib has a higher toxicity than nilotinib, i.e., $d_{anc,1} = 300/\text{mm}^3$ and $d_{anc,2} = 350/\text{mm}^3$. It usually takes less than a month for the ANC of a patient with grade 3-4 neutropenia to go back to normal level, so the monthly increase rate of ANC level is between 500 and 7000/mm [5]. In our model, we assume that the ANC of a patient increases by $b_{anc} = 2000/\text{mm}^3$ during a drug holiday, before it reaches the normal level 3000/mm^3.

B: Method to solve the optimization model

We describe the method to solve the optimization model introduced in the **Treatment optimization problem** section (see equations (2)-(6) of main text) with toxicity constraints given by equation (7) of the main text and $y^m \geq L_{anc}$ for $m \in \mathcal{M}$. Our strategy is to build a mixed-integer linear optimization model [8] that approximates the optimization model given by equations (2)-(6) of the main text, and then solve the approximation model to optimality numerically by off-the-shelf optimization software CPLEX [9]. The mixed-integer linear optimization model is built through two steps: (1) we first approximate the ODE constraints (equation (3) of main text) by bilinear constraints; (2) we then transform the bilinear constraints and nonlinear toxicity constraints (equation (7) of the main text) into equivalent linear constraints, by adding auxiliary decision variables.

We first describe how to approximate the ODE constraints (equation (3) of main text) by bilinear constraints. Suppose patients take drug j in month m. Since the cell dynamics are modeled by the following set of ODEs

$$\begin{aligned} \dot{x}(t) &= f^j(x(t)),\ t \in [m\Delta t, (m+1)\Delta t], \\ x(m\Delta t) &= x^m, \end{aligned}$$

the cell abundances in month $m+1$, x^{m+1}, are completely determined by the initial cell abundance x^m and function f^j. Without loss of generality, we assume this relationship is described by

$$x_{l,i}^{m+1} = g_{l,i}^j(x^m)$$

with some unknown nonlinear function $g_{l,i}^j : \mathbb{R}^{L\times n} \to \mathbb{R}$, for each month m, layer l, and cell type i. Recall that L is total number of cell layers ($L = 4$), and n is the total number of cell types. Then the ODE constraints (equation (3) of main text) are equivalent to the constraints below

$$x_{l,i}^{m+1} = \sum_{j\in\mathcal{J}} z^{m,j} g_{l,i}^j(x^m), \text{ for each } m, l, i. \tag{2}$$

We will approximate the nonlinear function $g_{l,i}^j$ with an affine function $\hat{g}_{l,i}^{m,j} : \mathbb{R}^{L\times n} \to \mathbb{R}$, for each m, j, l, and i. In particular, the function

$$\hat{g}_{l,i}^{m,j}(x) = a^{j,l,i}x + h_{l,i}^{m,j}, \tag{3}$$

where $a^{j,l,i}$ is an (Ln)-dimensional vector and does not depend on m. Details of how $\hat{g}_{l,i}^{m,j}$ is constructed are provided in Section "linear approximation to the solutions of the ODEs" of the Appendix. Let $a^{j,l,i} = [a_{1,1}^{j,l,i}, \ldots, a_{k,s}^{j,l,i}, \ldots, a_{L,n}^{j,l,i}]$. Then constraint (2) can be approximated by the bilinear constraint

$$x_{l,i}^{m+1} = \sum_{j\in\mathcal{J}} z^{m,j} \hat{g}_{l,i}^{m,j}(x^m) = \sum_{j\in\mathcal{J}} z^{m,j} (\sum_{k\in\mathcal{L}, s\in\mathcal{I}} a_{k,s}^{j,l,i} x_{k,s}^m + h_{l,i}^{m,j}), \tag{4}$$

for each type i cell at layer l in month m.

We now describe how to transform bilinear constraints (4) and piecewise linear constraints (equation (7) of the main text) into linear constraints. These are standard techniques in mixed-integer linear optimization [8]. We introduce auxiliary continuous variables $v_{k,s}^{m,j}$, and set $v_{k,s}^{m,j} = z^{m,j} x_{k,s}^m$. Then bilinear constraints (4) are transformed

into the equivalent linear constraints below.

$$\begin{aligned} x_{l,i}^{m+1} =& \sum_{j\in\mathcal{J}} (\sum_{k\in\mathcal{L},s\in\mathcal{I}} a_{k,s}^{j,l,i} v_{k,s}^{m,j} + h_{l,i}^{m,j} z^{m,j}) \\ 0 \le& v_{k,s}^{m,j} \le U_{k,s} z^{m,j}, \\ 0 \le& x_{k,s}^{m} - v_{k,s}^{m,j} \le U_{k,s}(1 - z^{m,j}), \end{aligned}$$

where $U_{k,s}$ is an upper bounds of cell abundance $x_{k,s}^{m}$ for each m. The value of $U_{k,s}$ can be obtained by taking the maximum value of layer k type s cell abundances over the whole planning horizon under all three monotherapies and no treatment. The piecewise linear constraints can be transformed into equivalent linear constraints below, by introducing auxiliary continuous variable u^m and binary variable q^m for each m.

$$\begin{aligned} u^{m+1} = y^m + b_{anc} z^{m,0} - \sum_{j\in\mathcal{J}\setminus\{0\}} d_{anc,j} z^{m,j}, \\ y^{m+1} \ge u^{m+1} - b_{anc} q^{m+1}, \\ y^{m+1} \ge U_{anc} - (U_{anc} - L_{anc})(1 - q^{m+1}), \\ y^{m+1} \le u^{m+1}, \\ y^{m+1} \le U_{anc}, \\ q^{m+1} \in \{0,1\}. \end{aligned}$$

Overall, the optimization model (equations (2)-(6) of main text) with toxicity constraints (equation (7) of main text and $y^m \ge L_{anc}$ for $m \in \mathcal{M}$) is approximated by the following mixed-integer linear optimization model.

$$\begin{aligned} \min\ & \sum_{l\ge1} \sum_{i\ge2} x_{l,i}^{M} \\ \text{s.t.}\ & x_{l,i}^{m+1} = \sum_{j\in\mathcal{J}} \sum_{k\in\mathcal{L},s\in\mathcal{I}} a_{k,s}^{j,l,i} v_{k,s}^{m,j} + \sum_{j\in\mathcal{J}} h_{l,i}^{m,j} z^{m,j},\ i \in \mathcal{I}, l \in \mathcal{L}, m \in \mathcal{M}\setminus\{M\} \\ & 0 \le v_{k,s}^{m,j} \le U_{k,s} z^{m,j},\ i \in \mathcal{I}, l \in \mathcal{L}, m \in \mathcal{M}\setminus\{M\} \\ & 0 \le x_{k,s}^{m} - v_{k,s}^{m,j} \le U_{k,s}(1 - z^{m,j}),\ i \in \mathcal{I}, l \in \mathcal{L}, m \in \mathcal{M}\setminus\{M\} \\ & u^{m+1} = y^m + b_{anc} z^{m,0} - \sum_{j\in\mathcal{J}\setminus\{0\}} d_{anc,j} z^{m,j},\ m \in \mathcal{M}\setminus\{M\} \\ & y^{m+1} \ge u^{m+1} - b_{anc} q^{m+1},\ m \in \mathcal{M}\setminus\{M\} \\ & y^{m+1} \ge U_{anc} - (U_{anc} - L_{anc})(1 - q^{m+1}),\ m \in \mathcal{M}\setminus\{M\} \\ & y^{m+1} \le u^{m+1},\ m \in \mathcal{M}\setminus\{M\} \\ & y^{m+1} \le U_{anc},\ m \in \mathcal{M}\setminus\{M\} \\ & y^m \ge L_{anc},\ m \in \mathcal{M} \\ & \sum_{j\in\mathcal{J}} z^{m,j} = 1,\ m \in \mathcal{M}\setminus\{M\} \\ & z^{m,j}, q^{m+1} \in \{0,1\},\ m \in \mathcal{M}\setminus\{M\}, j \in \mathcal{J} \\ & x(0) = x^0,\ y^0 \text{ is given.} \end{aligned} \tag{6}$$

C: Linear approximation to the solutions of the ODEs

In this section, we describe how to construct the affine function $\hat{g}_{l,i}^{m,j}$ in (3) in Section "Method to solve the optimization model" of the Appendix. If we assume that the drugs

do not affect stem cells, we can compute the abundance of stem cells over the planning horizon numerically in advance, regardless of the treatment schedules. Thus we assume $x_{1,1}(t), \ldots, x_{1,n}(t)$ are given as data, for any t. We can first eliminate all the variables $x^m_{1,i}$ and constraints containing $x^m_{1,i}$, for each m and i, in the optimization problem (6). The dynamics of wild-type leukemic cells and each mutant type have no impact on each other. We can decouple the ODEs into a series of linear ODEs as follows, each describing the dynamics for type i cell from layer 2 to layer 4.

$$\begin{bmatrix} \dot{x}_{2,i}(t) \\ \dot{x}_{3,i}(t) \\ \dot{x}_{4,i}(t) \end{bmatrix} = \begin{bmatrix} -d^j_{2,i} & 0 & 0 \\ b^j_{3,i} & -d^j_{3,i} & 0 \\ 0 & b^j_{4,i} & -d^j_{4,i} \end{bmatrix} \begin{bmatrix} x_{2,i}(t) \\ x_{3,i}(t) \\ x_{4,i}(t) \end{bmatrix} + \begin{bmatrix} b^j_{2,i} x_{1,i}(t) \\ 0 \\ 0 \end{bmatrix} \tag{7}$$

Write the above equations in the matrix form, we have

$$\dot{v}_i(t) = W^j_i v_i(t) + w^j_i(t), \text{ for } t \in [m\Delta t, (m+1)\Delta t], \tag{8}$$

where $v_i(t) = [x_{2,i}(t), x_{3,i}(t), x_{4,i}(t)]^\top$, $w^j_i(t) = [b^j_{2,i} x_{1,i}(t), 0, 0]^\top$, and W^j_i is the lower triangular matrix in (7).

We divide $(m\Delta t, (m+1)\Delta t)$ into $\Delta t = 30$ one-day sub-intervals. Consider a sub-interval $(t_0, t_0 + 1)$. By assuming $w^j_i(t) = w^j_i(t_0)$ for any $t \in (t_0, t_0 + 1)$, we solve (8) approximately and obtain

$$v_i(t_0 + 1) \approx e^{W^j_i} v_i(t_0) + (e^{W^j_i} - I)(W^j_i)^{-1} w^j_i(t_0). \tag{9}$$

By combining equations (9) for $t_0 = m\Delta t, m\Delta t + 1, \ldots, (m+1)\Delta t - 1$, we have

$$v_i((m+1)\Delta t) = e^{W^j_i \Delta t} v_i(m\Delta t) + \sum_{d=0}^{\Delta t - 1} e^{W^j_i (\Delta t - 1 - d)} (e^{W^j_i} - I)(W^j_i)^{-1} w^j_i(m\Delta t + d). \tag{10}$$

Recall that $v_i(m\Delta t) = [x^m_{2,i}, x^m_{3,i}, x^m_{4,i}]^\top$ for each m. Thus (10) can be rewritten as

$$\begin{bmatrix} x^{m+1}_{2,i} \\ x^{m+1}_{3,i} \\ x^{m+1}_{4,i} \end{bmatrix} = A^{j,i} \begin{bmatrix} x^m_{2,i} \\ x^m_{3,i} \\ x^m_{4,i} \end{bmatrix} + h^{m,j}_i, \tag{11}$$

where $A^{j,i} = (e^{W^j_i})^{\Delta t}$ and $h^{m,j}_i = \sum_{d=0}^{\Delta t - 1} (e^{W^j_i})^{\Delta t - 1 - d} (e^{W^j_i} - I)(W^j_i)^{-1} w^j_i(m\Delta t + d)$. Each equation in (11) is used as the affine function $\hat{g}^{m,j}_{l,i}$ in (3), for each m, j, i, and $l = 2, 3, 4$.

References

1. Olshen A, Tang M, Cortes J, Gonen M, Hughes T, Branford S, et al. Dynamics of chronic myeloid leukemia response to dasatinib, nilotinib, and high-dose imatinib. Haematologica. 2014;99(11):1701–1709.

2. Redaelli S, Piazza R, Rostagno R, Magistroni V, Perini P, Marega M, et al. Activity of bosutinib, dasatinib, and nilotinib against 18 imatinib-resistant BCR/ABL mutants. Journal of Clinical Oncology. 2009;27(3):469–471.

3. Dingli D, Traulsen A, Pacheco JM. Chronic myeloid leukemia: origin, development, response to therapy, and relapse. Clinical Leukemia. 2008;2(2):133–139.

4. Holyoake TL, Jiang X, Drummond MW, Eaves AC, Eaves CJ. Elucidating critical mechanisms of deregulated stem cell turnover in the chronic phase of chronic myeloid leukemia. Leukemia. 2002;16(4):549–558.

5. Kantarjian H, Sawyers C, Hochhaus A, Guilhot F, Schiffer C, Gambacorti-Passerini C, et al. Hematologic and cytogenetic responses to imatinib mesylate in chronic myelogenous leukemia. New England Journal of Medicine. 2002;346(9):645–652.

6. Cortes JE, Jones D, O'Brien S, Jabbour E, Konopleva M, Ferrajoli A, et al. Nilotinib as front-line treatment for patients with chronic myeloid leukemia in early chronic phase. Journal of Clinical Oncology. 2010;28(3):392–397.

7. Cortes JE, Saglio G, Kantarjian HM, Baccarani M, Mayer J, Boqué C, et al. Final 5-Year study results of DASISION: the dasatinib versus imatinib study in treatment-naïve chronic myeloid leukemia patients trial. Journal of Clinical Oncology. 2016;34(20):2333–2340.

8. Nemhauser GL, Wolsey LA. Integer and combinatorial optimization. John Wiley & Sons; 1999.

9. IBM. IBM ILOG CPLEX: high-performance mathematical programming engine; 2015. http://www-01.ibm.com/software/in/integration/optimization/cplex/.
